# Evaluation of serum vitamin b12 in elderly individuals with type 2 diabetes mellitus, with and without dementia, in relation to the use of metformin

**DOI:** 10.1186/1758-5996-7-S1-A105

**Published:** 2015-11-11

**Authors:** Danilo Hojo Navarro, Marcelo d'Oliveira, Tali Wajsfeld, João Eduardo Nunes Salles

**Affiliations:** 1 Faculdade de Ciências Médicas da Santa Casa de São Paulo, São Paulo, Brazil

## Background

Diabetes mellitus (DM) is a group of metabolic diseases with diverse pathophysiology, characterized by hyperglycemia resulting from impaired insulin secretion by beta cells, peripheral resistance to insulin action, or both. One of the ways to treat diabetes is using Metformin, which reduces the blood glucose by suppressing hepatic glucose production, increasing muscle glucose uptake and insulin-dependent increase intestinal glucose utilization. But like any drug metformin also has side effects and what interests us in this work is to study the chronic treatment with metformin, which may be accompanied by a slight decrease absorption of vitamin B12 in the distal ileum and occasionally folate. Studies have identified the existence of a connection between Diabetes Mellitus and dementia. Hyperglycemia can be a significant factor for the incidence of Alzheimer's and a secondary cause of dementia. The Type 2 Diabetes Mellitus is associated with cognitive and functional deficits. One of the Required laboratory test in the etiological investigation of a dementia syndrome is the serum levels of vitamin B12. Under these conditions, the diagnosis is based largely on clinical history, as well as the neuropsychological profile.

## Objective

Through cross-sectional evaluate the influence of Metformin in elderly patients with and without dementia, on the levels of vitamin B12 METHOD Study across 110 elderly dementia patients from the discipline of neurology at the Santa Casa de São Paulo and 100 of ambulatory diabetes, where we analyzed and compared the progression of dementia and values of B12.

## Results

Figure 1.

**Figure 1:**
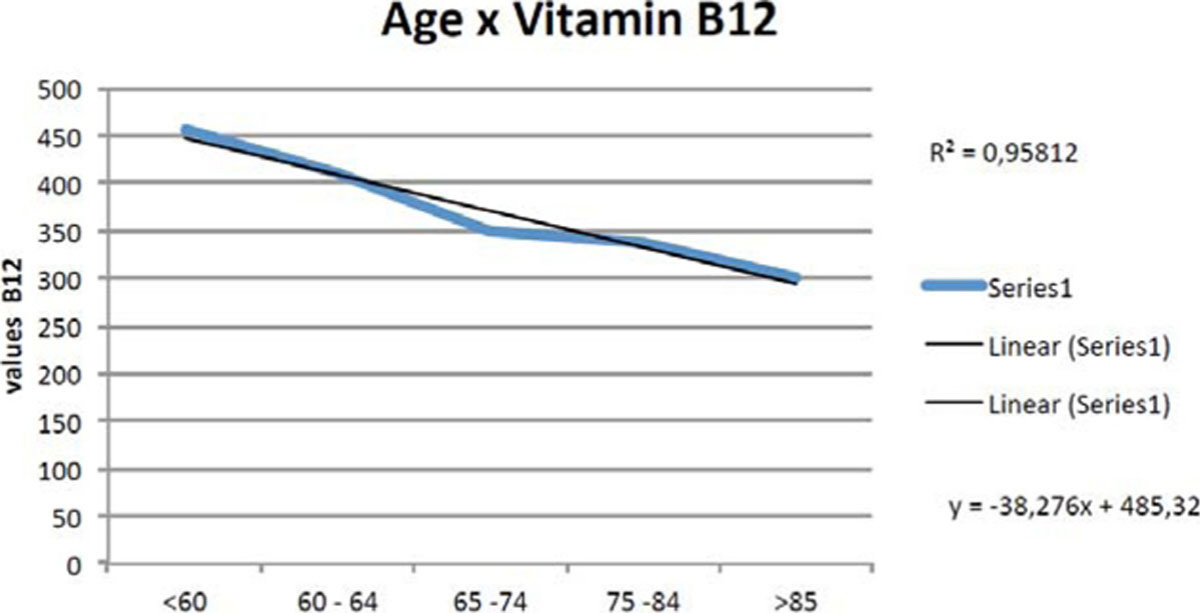
Results showing values of vitamin B12 in relation to age.

## Discussion and conclusion

According to the results, we noticed and we proved that the Metformin influenced on reducing the amounts of vitamin B12 and when we compare the values of vitamin B12 in relation to age of all patients being diabetics or those with cognitive impairment, we found values with great statistical significance, demonstrating a linear decreases Vitamin B12 according to the increasing age of the individual. Also the value of Chi-Square close to one emphasizes that there is a tendency in the study population and not a finding at random. The hypothesis of a dementia related to diabetes as a secondary cause is a doubt as the majority of patients uses metformin, which decreases the amounts of B12 which is included in laboratory research on dementia.

